# Evidence for Environmental Dissemination of Antibiotic Resistance Mediated by Wild Birds

**DOI:** 10.3389/fmicb.2018.00745

**Published:** 2018-04-20

**Authors:** Jiao Wu, Ye Huang, Dawei Rao, Yongkui Zhang, Kun Yang

**Affiliations:** Department of Pharmaceutical & Biological Engineering, School of Chemical Engineering, Sichuan University, Chengdu, China

**Keywords:** antibiotic resistance, environmental dissemination, wild birds, antibiotic resistance gene, horizontal gene transfer

## Abstract

The aquatic bird, egret, could carry antibiotic resistance (AR) from a contaminated waterway (Jin River, Chengdu, China) into the surrounding environment (Wangjianglou Park). A systematic study was carried out on the unique environmental dissemination mode of AR mediated by birds. The minimum inhibitory concentrations of various antibiotics against the environmental *Escherichia coli* isolates were used to evaluate the bacterial AR at the environmental locations where these isolates were recovered, i.e., the Jin River water, the egret feces, the park soil, and the campus soil. The level of AR in the park soil was significantly higher than that in the campus soil that was seldom affected by the egrets, which suggested that the egrets mediated the transportation of AR from the polluted waterway to the park. Genotyping of the resistant *E. coli* isolates via repetitive-element PCR gave no strong correlation between the genotypes and the AR patterns of the bacteria. So, the transfer of resistant strains should not be the main mode of AR transportation in this process. The results of real-time PCR revealed that the abundance of antibiotic resistance genes (ARGs) and mobile genetic element (MGE) sequences (transposase and integrase genes) declined along the putative transportation route. The transportation of ARGs could be due to their linkage with MGE sequences, and horizontal gene transfer should have contributed to the process. The movable colistin-resistance gene *mcr-1* was detected among the colistin-resistant *E. coli* strains isolated from the river water and the egret feces, which indicated the possibility of the environmental dissemination of this gene. Birds, especially the migratory birds, for the role they played on the dissemination of environmental AR, should be considered when studying the ecology of AR.

## Introduction

The problem of environmental antibiotic resistance (AR) should be recognized from three levels. First, the discharge of antibiotics at sub-inhibition dosage raised from anthropogenic activities exerts long-standing selective pressure on environmental microbial community. Second, resistant bacteria may keep proliferating, disseminating, and persisting in environment. Third, antibiotic resistance genes (ARGs) transport among bacteria of different species via horizontal gene transfer (HGT) and persist in environmental microbial community. For the first level, we have already realized that it is necessary to restrict the environmental emission of antibiotics. Various methods have been developed for the detection of antibiotics in environment (Hirsch et al., 1999; Seifrtova et al., 2008; Yang et al., 2010), and the methods for efficiently eliminating antibiotics from polluted environments are also under development (Ji et al., 2009; Homem and Santos, 2011; Peterson et al., 2012; Fukahori et al., 2013; Bao et al., 2014; Shi et al., 2014; Zuo et al., 2016). But for the second and third levels, i.e., the dissemination of AR in environment and its intrinsic mechanism are not yet fully understood, and effective methods for controlling the dissemination need to be developed. The dominant mechanisms for AR transportation under certain environmental conditions are still unclear. Is it the transfer of resistant bacteria, the horizontal transfer of ARGs or even both of them that governs the transportation process? The question has not yet been answered. The scope and extent of the environmental AR pollution is also far from being fully understood.

Although the exact mechanism is not yet fully understood, most of the existing research results have revealed that the dissemination of environmental AR is closely related to anthropogenic factors. Bacteria seldom develop AR at regions free from antibiotic pressure and human activities (Thaller et al., 2010). The environmental discrepancies originated from anthropogenic factors structured the community differences in resistant bacteria of different regions (Skurnik et al., 2006). Nevertheless, Cristóbal-Azkarate et al. (2014) isolated *Escherichia coli* strains with resistance to clinic synthetic or semi-synthetic antibiotics from the feces of wildlife at northeast of Mexico. Bhullar et al. (2012) found in an isolated cave at New Mexico bacteria with high AR levels. Some strains were even resistant to 14 different commercially available antibiotics. All the information has indicated that the AR and its dissemination have already exceeded previous anticipations. More and more facts support the growing opinion that AR is a natural and ancient character of microbial community, and environmental microorganisms are reservoirs of ARGs (Allen et al., 2009; Forsberg et al., 2012). Meanwhile, scientists also have speculated that migratory animal, especially the migratory birds may carry resistant bacteria or genes and transport them to regions far from anthropogenic influences (Allen et al., 2010). The Enterobacteriaceae strains isolated from migratory birds feces on the island of Ustica, Sicily (Italy) showed high proportions of resistance against ampicillin, amoxicillin–clavulanic acid, and streptomycin (Foti et al., 2011). The migratory birds of prey at Germany and Mongolia carried similar proportion of extended-spectrum beta-lactamase (ESBL)-producing *E. coli* (Germany: 13.8%, Mongolia: 10.8%; Guenther et al., 2012). The dissemination of environmental AR mediated by the activities of birds, especially the migratory birds, has been gradually recognized (Dolejska et al., 2007, 2009; Literak et al., 2007; Poeta et al., 2008; Radimersky et al., 2010; Radhouani et al., 2012). Birds can be potential spreaders of environmental AR. Their activities can mediate remote transportation of environmental AR, interconnect environmental locations of different AR levels and patterns, and even bring the AR to unfrequented places. Billions of birds travel between their winter homes and summer breeding grounds each year, and the area of their migration covers all continents including Antarctica (Yogui and Sericano, 2009; Muller et al., 2016). As a special dissemination mode of AR in the environment, the bird-mediated transportation of AR and its intrinsic mechanism has not yet been systematically studied.

China is not only the biggest producer but also the largest consumer of antibiotics in the world (Zhang et al., 2015). Both antibiotics and bacterial AR have been routinely detected in different geographic regions of China (Xu et al., 2007; Luo et al., 2010; Tao et al., 2010; Yang et al., 2010; Su et al., 2011, 2012; Wei et al., 2011; Zou et al., 2011; Zhu et al., 2013; Xiong et al., 2015). The watershed (Jin River) at Chengdu suffered persistent pollution of AR from both suburban and urban sources according to our recent survey (data not shown). Dozens of egrets inhabit the river, drink and forage in the river; while at night, they reside in a park (Wangjianglou Park) nearby the river. It is reasonable to assume that the AR can be transported from the river to the park through the activities of these egrets. To prove this and try to find the intrinsic mechanism of the AR transportation therein, systematic study was carried out on this specific dissemination mode of environmental AR mediated by the activities of these egrets. The *E. coli* strains were isolated from the environmental samples nearby the habitation of egrets and the AR level was assessed by determining their antibiotic minimum inhibitory concentrations (MICs). By comparing the level of bacterial AR from different environmental locations, the direction and route of resistance transportation were determined. The genotypes and AR patterns of the *E. coli* isolates were analyzed in combination to disclose the intrinsic mechanism of the resistance transportation (the transfer of resistant bacteria or the horizontal transfer of resistance genes). The abundances of ARGs and mobile genetic element (MGE) sequences (transposase and integrase genes) in various environmental locations were determined via real-time PCR. The role of HGT on the environmental dissemination of AR was thus verified. The environmental dissemination of the newly discovered movable colistin resistance gene, *mcr-1*, was investigated via PCR survey of this gene among colistin-resistant *E. coli* isolates.

## Materials and Methods

### Sampling

**FIGURE 1:**
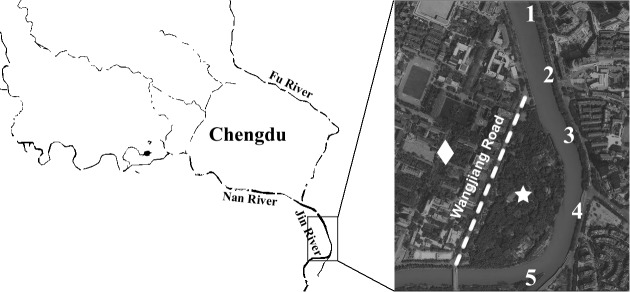
Illustration of sampling efforts. The numbers along the river marked the sites of water sampling. The star indicates the position of Wangjianglou Park, while the diamond indicates the Wangjiang campus of Sichuan University. The dashed line labels the Wangjiang Road that separates the park and the campus.

Water samples (500 mL each) were collected from five sampling sites along the Jin River (**Figure 1**) on January 21, March 13, March 26, and April 1 of 2015, respectively. The GPS coordinates of the five sampling sites are as follows: 30° 38′ 23.8″ N and 104° 5′ 20.9″ E for site 1, 30° 38′ 13.1″ N and 104° 5′ 25.4″ E for site 2, 30° 38′ 3.4″ N and 104° 5′ 29″ E for site 3, 30° 37′ 52.6″ N and 104° 5′ 31″ E for site 4, and 30° 37′ 44″ N and 104° 5′ 21.8″ E for site 5. On the same sampling day, five soil samples were also collected from the nightly inhabit of the egrets (Wangjianglou Park) at the GPS coordinate around 30° 37′ 50.3″ N and 104° 5′ 24.4″ E. Additionally, five samples of fresh feces-droppings of egrets were collected in the same park on April 27, 2015. Five soil samples for control were collected on the campus of Sichuan University on September 7, 2015. The campus is separated by a road (the Wangjiang Road) from the park and is seldom affected by egrets. The GPS coordinates of five sampling sites on the campus are as follows: 30° 37′ 54″ N and 104° 4′ 34″ E for sample 1, 30° 37′ 54″ N and 104° 4′ 43″ E for sample 2, 30° 38′ 0″ N and 104° 4′ 48″ E for sample 3, 30° 37′ 57″ N and 104° 4′ 49″ E for sample 4, and 30° 38′ 1″ N and 104° 5′ 1″ E for sample 5. All samples were treated within 5 h after collection.

### *Escherichia coli* Isolation

Solid samples (soil or feces) were first extracted using sterile distilled water to release *E. coli* cells in a procedure based on a previously described technique (Boehm et al., 2009; Cui et al., 2013). One milliliter of serial 10-fold dilutions of the solid sample extracts (10^-1^ and 10^-2^ for soil samples, and from 10^-2^ to 10^-4^ for feces samples) or the water samples (1, 10^-1^, and 10^-2^) were filtered through sterile 0.45 μm S-Pak^®^ membranes (Millipore, Billerica, United States). For homogenous distribution of the bacteria, 20 mL of sterilized distilled water was applied to the membrane before exerting vacuum. The cell-bearing membranes were transferred onto membrane-Thermotolerant *E. coli* (mTEC) agar to selectively grow *E. coli* (USEPA, 2002).

Presumptive *E. coli* colonies were randomly picked from mTEC agar and streaked on Luria-Bertani (LB) agar for purification. After single colonies were picked from streak plates and inoculated in LB broth, the authenticity of the *E. coli* isolates was then verified by indole–methyl red–Voges–Proskauer–citrate (IMViC) tests, which resulted in verification ratios higher than 90%. The verified *E. coli* isolates were stored as glycerol stocks at -80°C for subsequent analysis.

### Assessment of Antibiotic Resistance of Environmental *E. coli* Isolates

The MICs of 11 antibiotics against the *E. coli* isolates were determined in order to assess their AR. MIC data were collected via a modified broth micro-dilution method (Andrews, 2001). The *E. coli* isolates were first grown in 96-well plates with LB broth at 37°C overnight to reach stationary phase. The cell cultures were then used to inoculate test plates that contained LB broth with a range of antibiotic concentrations (0.25, 0.5, 1, 2, 4, 8, 16, 32, 64, and 128 μg/mL). For the two antibiotics with higher antimicrobial potencies, ceftriaxone and ciprofloxacin, the range of concentrations in the broth was set at 0.0125, 0.025, 0.05, 0.1, 0.2, 0.4, 0.8, 1.6, 3.2, and 6.4 μg/mL. The inoculation was executed with a flame-sterilized 48-pin replicator to ensure the uniform inoculum density. The MIC endpoints were determined as the lowest concentration at which there was no visible growth after 20 h of incubation at 37°C. Duplicate tests for each antibiotic concentration were conducted, and the average number was calculated as the MIC value. Positive and negative controls were conducted in antibiotic-free LB to ensure growth of environmental *E. coli* isolates under lab conditions and sterility of the assay, respectively. Quality control of the procedure was conducted by using the susceptive *E. coli* standard strain ATCC 25922, which exhibited similar antibiotic MIC values as reported in the literature (Andrews, 2001).

Minimum inhibitory concentration breakpoint for each antibiotic was set at 16 times of the MIC of the standard strain. The isolates with MICs greater than the breakpoint were considered resistant. The AR level against an antibiotic was represented with corresponding percentage of resistant strains. The multiple antibiotic resistance index (MARI) was calculated to evaluate the multidrug resistance (MDR) for each type of environmental sample (Krumperman, 1983).

$$MARI=\Sigma_{i=1}^{c}a_{i}/\left(b\times c\right)$$

Where *c* is the number of individual strains isolated from specific type of environmental sample, *a_i_* is the number of antibiotics that strain *i* is resistant to, *b* is the number of antibiotics tested (11 in our case).

In order to compare the AR levels between different environmental locations, the antibiotic MIC50 and MIC90 values of the *E. coli* isolates from different environmental locations were normalized against the MIC values of the standard strain ATCC 25922, and thereafter were log_2_-transformed. The percentages of resistant strains against each antibiotic at different environmental locations were also log-transformed. The transformed MIC50/MIC90 values and resistance percentages against 11 antibiotics at two different environmental locations can be looked as pairs of observations. The paired-sample *t*-test was used to determine whether the difference in AR level at the two environmental locations was significant. A *p*-value of less than 0.05 was considered statistically significant.

In order to analyze the AR pattern of the *E. coli* isolated from different environmental samples, the MIC data were firstly normalized against the corresponding MICs of the standard strain. Then, the normalized MICs were multiplied with 100 and thereafter log_2_-transformed. The transformed MICs data were treated as a series of multidimensional vectors that represent the drug resistance patterns of the *E. coli* isolates. The transformed MICs were used to calculate the Euclidean distances between different strains. The generated Euclidean distance matrix was thereafter subjected to non-metric multidimensional scaling (NMDS) to compare the AR patterns of the *E. coli* isolated from different environmental samples. The NMDS was performed using 100 random starting configurations of sample points (*E. coli* isolates) with the built-in midscale function of Matlab_2016Ra (MathWorks, Natick, MA, United States); the accuracy of the NMDS representation was determined by calculating the Kruskal stress (Cui et al., 2013). A contour map of MDR was generated via interpolating the MDR data of these *E. coli* isolates using the scatteredInterpolant function of Matlab_2016Ra (MathWorks, Natick, MA, United States) and lined behind the NMDS plot.

### Genetic Diversity of Environmental *E. coli* Isolates

Genomic DNA fingerprinting of *E. coli* isolates was performed using repetitive-element PCR (rep-PCR; Dombek et al., 2000). Briefly, fresh *E. coli* cells were collected via centrifugation from overnight 96-well plate pure culture in LB broth and then gently treated (60°C for 20 min) with 0.05 N NaOH to release total genomic DNA. The total genomic DNA in solution was separated from cell debris by centrifugation at 250 rpm for 10 min. The supernatants containing total genomic DNA were used as templates in PCR amplification for rep-PCR DNA fingerprinting. The BOX-A1R primer (5′-CTACGGCAAGGCGACGCTGACG-3′) was used in this study. Following amplification, the PCR amplicons were electrophoresed, and the gel images were obtained using a Junyi gel imaging system JY04S-3E (Beijing Junyi Dongfang Electrophoresis Co., Ltd., Beijing). The DNA banding pattern (i.e., fingerprint) for each isolate was normalized for inter-gel comparisons using an external DNA size marker (100 bp DNA Marker; Beijing Dingguo Changsheng Biotechnology Co., Ltd., Beijing) that was loaded into both end and middle lanes of each gel. All fingerprint images were loaded into a database and processed using BioNumerics (Applied Maths, Kortrijk, Belgium). The dendrogram was created based on Pearson’s correlation using unweighted-pair group method with arithmetic means (UPGMA).

### Quantify the Abundance of ARGs and MGEs by Real-Time PCR (qPCR)

In order to quantify the abundance of ARGs and MGEs, we intensively collected the environmental samples once again in September 2015. The river water, egret feces, park soil, and campus soil were sampled in triplicate, respectively. Solid samples (soil and egret feces, 1 g each) and filtration retentates of water samples (from about 300 mL water sample each filtered through GN-6 0.45 μm membranes) were extracted the total genomic DNA with the FastDNA Spin Kit for Soil (MP Biomedicals, LLC., United States). DNA extracts of the same sample type were pooled together to make four composite DNA samples representing Jin River water, egret feces, park soil, and campus soil, respectively. The DNA samples were used as templates in qPCR to evaluate the abundances of ARGs and MGEs therein. The 16S rRNA gene was used as reference standard. All qPCRs were operated in triplicate. Quality control was obtained via checking the melting curves and electrophoresis image of the qPCR products after amplification. Confirmed specific amplifications were thereafter executed the data analysis. The abundances of ARGs and MGEs were expressed as the amount ratios of target genes to the reference gene. The acquisition of primer pairs, qPCR operation, and related data treatments were illustrated in Appendix 1 in Supplementary Material. The information of the target genes and related primer pairs were summarized in Supplementary Table S2. Averages, standard deviations, and relative abundance of ARGs and MGEs were determined using Excel 2013 (Microsoft Office 2013, Microsoft, United States). The clustered heatmap was performed in Matlab_2016Ra (MathWorks, Natick, MA, United States).

### Survey of *mcr-1* Gene Among Colistin-Resistant *E. coli* Isolates

Some colistin-resistant *E. coli* isolates (*n* = 6) were recovered from environmental samples in our case. Among these isolates, four were isolated from the river water, one from the egret feces and the other one from the park soil. Consequently, a PCR survey targeting *mcr-1* gene among these colistin-resistant *E. coli* isolates was executed with the primer pair CLR5-F (5′-CGGTCAGTCCGTTTGTTC-3′) and CLR5-R (5′-CTTGGTCGGTCTGTAGGG-3′) (Liu et al., 2016). The PCR amplification products were sent to Sangon Biotech (Shanghai, China) for sequencing.

## Results

### Bacterial Antibiotic Resistance in Different Environmental Samples

**FIGURE 2:**
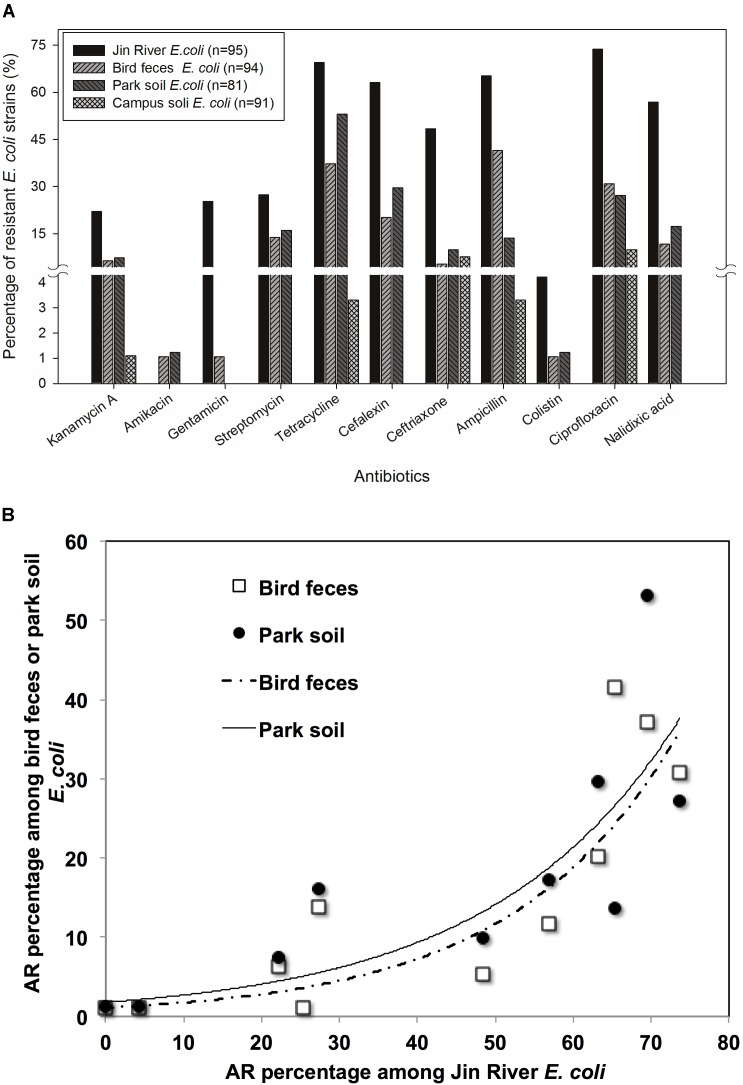
The role of bird in the AR transportation was indicated with the AR rates (percentage) of environmental *E. coli* isolates **(A)** and the correlation of AR rates among the *E. coli* strains isolated from the environmental samples in related to egret inhabitation **(B)**. According to the paired-sample *t*-test of the AR rates (log-transformed) between the *E. coli* strains isolated from different environmental samples, the *E. coli* strains isolated from Jin River water exhibited the highest resistance rates to 11 antibiotics (*p* < 0.01). The campus soil isolates gave the lowest resistance rates (*p* < 0.01). The AR rates of the egret feces isolates were similar to those of the park soil isolates **(A)**. The resistance rates of the bird feces and park soil isolates to 11 antibiotics showed good exponential correlations (*R*^2^ > 0.77, *p* < 0.01) with those of the Jin River water isolates **(B)**.

Ninety-five *E. coli* isolates were recovered from river water, 94 from egret feces, 81 from park soil, and 91 from campus soil. The information of the *E. coli* collection was summarized in Supplementary Table S1. MICs data of 11 antibiotics against these *E. coli* isolates indicated the bacterial AR levels in different environmental samples. MIC50 and MIC90 of each group of *E. coli* strains isolated from different environmental samples (**Table 1**) showed that the AR level in Jin River water was the highest (paired-sample *t*-test, *p* < 0.05). The lowest level of AR occurred in the campus soil (*p* < 0.01). The AR in egret feces and park soil was at a similar level. The percentages of resistant *E. coli* among different environmental samples followed the same trend (**Figure 2A**). The *E. coli* strains isolated from Jin River water exhibited the highest resistance rates to 11 antibiotics (*p* < 0.01). The campus soil isolates gave the lowest resistance rates (*p* < 0.01). The resistance rates of the egret feces isolates to these antibiotics were similar to those of the park soil isolates. The resistance rates of the bird feces and park soil isolates to 11 antibiotics showed good exponential correlations with those of the Jin River water isolates, as demonstrated in **Figure 2B** (*R*^2^ > 0.77, *p* < 0.01). The detailed MIC distributions of the *E. coli* isolates from four environmental sample types are summarized in Supplementary Tables S3–S6, respectively. The antibiotic MICs of the susceptible standard *E. coli* strain, ATCC 25922, were also listed in the last column of Supplementary Table S3.

**Table 1:** MIC50 and MIC90 of different antibiotics against the *E. coli* strains isolated from different environmental samples.

	MIC50/MIC90 (μg/mL)			
	
Antibiotics	Jin River *E. coli* isolates (*n* = 95)	Egret feces *E. coli* isolates (*n* = 94)	Park soil *E. coli* isolates (*n* = 81)	Campus soil *E. coli* isolates (*n* = 91)
Kanamycin A	32/>128	32/64	16/32	16/32
Amikacin	8/16	32/32	16/32	16/16
Gentamicin	4/>128	8/8	4/6	4/8
Streptomycin	32/>128	32/>128	32/>128	16/16
Tetracycline	64/>128	2/128	>128/>128	1/1
Cefalexin	>128/>128	64/>128	128/>128	32/64
Ampicillin	128/>128	4/>128	16/>128	2/4
Colistin	6/16	2/4	0.25/2	1/2
Nalidixic acid	64/>128	2/16	4/>128	16/16
Ceftriaxone	1/>6.4	0.1/0.2	0.025/1	0.025/0.025
Ciprofloxacin	>6.4/>6.4	0.0125/0.4	0.2/>6.4	0.025/0.175


**According to the paired-sample *t*-test of the MIC90 values, the AR level in Jin River is significantly the highest (*p* < 0.05), while that in campus soil is the lowest (*p* < 0.01). The AR in egret feces and park soil is at a similar level.**

**FIGURE 3:**
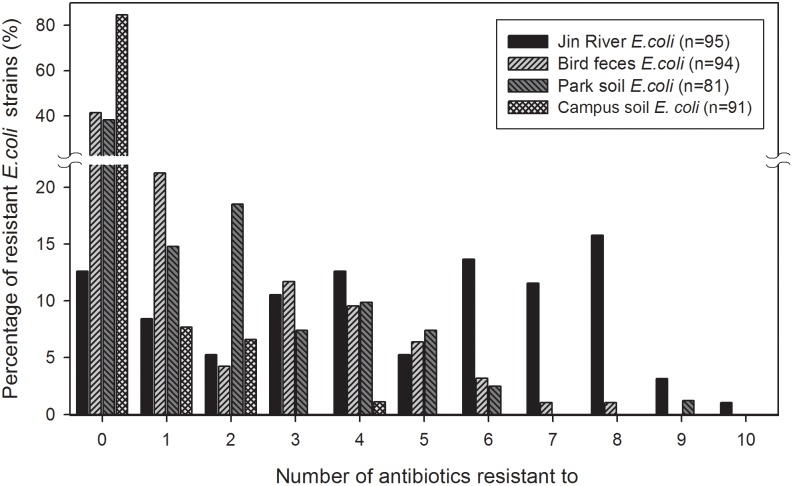
Multidrug resistance (MDR) among the *E. coli* isolates from different environmental samples. The *E. coli* isolates of Jin River water gave the highest level of MDR. Most of the campus soil isolates (92.3%) showed no MDR. The *E. coli* isolated from egret feces and park soil exhibited similar level of MDR.

The MDR of these *E. coli* isolates are recorded and exhibited in **Figure 3**. The MARIs were calculated for four types of environmental samples and listed in **Table 2**. The situation of MDR was the most serious among the *E. coli* strains isolated from Jin River water, and followed by the strains isolated from egret feces and park soil. Most of the campus soil isolates (92.3%) exhibited no MDR.

**Table 2:** Multiple antibiotic resistance indexes (MARIs) of environmental samples.

Environmental samples (No. of *E. coli* isolates)	Jin River water (*n* = 95)	Egret feces (*n* = 94)	Park soil (*n* = 81)	Campus soil (*n* = 91)
MARI	0.414	0.155	0.160	0.023


**FIGURE 4:**
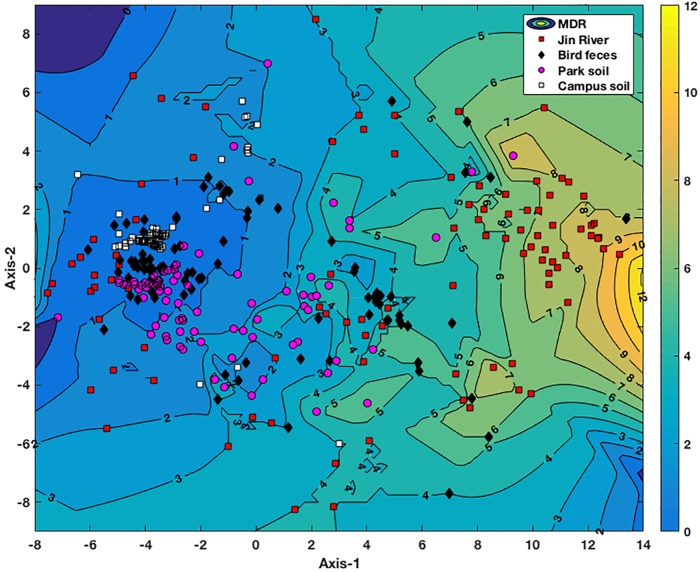
NMDS analysis of antibiotic resistance pattern of the *E. coli* isolates based on their antibiotic MIC data. The Kruskal stress is 0.1273. A contour map of multidrug resistance (MDR) of the *E. coli* isolates is generated via interpolating the MDR data of these strains and lined behind the NMDS plot. The numbers on the contour lines are the numbers of antibiotics that one strain is resistant to. The degree of the MDR is indicated by a color bar with warm color (yellow) representing high level of MDR and cool color (blue) for low MDR degree.

Non-metric multidimensional scaling plot based on the MIC data of the *E. coli* isolates is exhibited in **Figure 4**. The *E. coli* strains isolated from different environmental samples exhibited various patterns of AR and MDR. The *E. coli* group recovered from Jin River water (red squares) contained strains of various AR patterns, which indicated their “multiple sources.” On the other hand, both of the *E. coli* groups isolated from egret feces (black diamonds) and park soil (pink dots) contained either strains of low-level AR or strains of various AR patterns with high degree of MDR. The *E. coli* group isolated from campus soil (white squares), however, exhibited mostly the low-level AR. Only few isolates gave weak MDR (resistant to two to four antibiotics).

### Genetic Diversity of the *E. coli* Isolates

Genetic diversity of environmental *E. coli* isolates was reflected via rep-PCR fingerprinting. According to the dendrogram of the DNA banding patterns (Supplementary Figure S1), most of the bacterial clusters of similar genotypes occurred among the *E. coli* strains isolated from the same environmental sample, while only minority included the isolates from different samples. At the threshold of 90% similarity in genomic structures (rep-PCR banding patterns), several typical clusters of *E. coli* isolates recovered from the same (F, G, and H) or different (A, B, C, D, and E) type of environmental samples were recorded the AR patterns of their members. The results are summarized in Supplementary Table S7.

### Abundance of ARGs and MGEs in Environment

**FIGURE 5:**
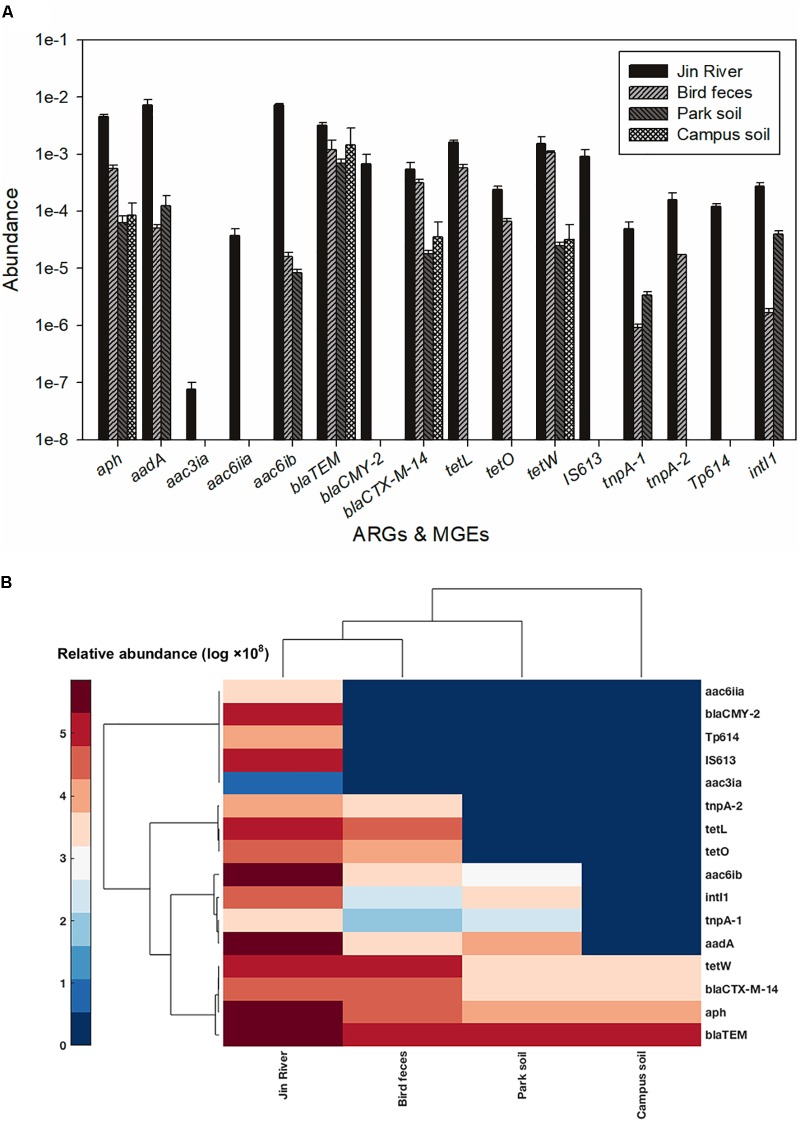
Abundance of ARGs and MGEs among different environmental samples **(A)** and the heatmap of the abundance (log-transformed) of ARGs and MGEs **(B)**. The legend for the heatmap denotes corresponding log-transformed values of the abundances of these genes. Both columns (samples) and rows (genes) were clustered based on cosine similarity. None of the five MGEs was detected in the campus soil.

The result of AR assessment via qPCR coincided fairly well with that obtain via culture-based method (MIC data). A typical fact is that at the location where the phenotypes of resistance against certain antibiotics (tetracycline or cephalosporin) were detected (**Figure 2A**), the corresponding ARGs (*tetW* or *bla*TEM and *bla*CTX-M-14, respectively) were also mostly detected therein (**Figure 5A**). The transportation of different ARGs varied from one to another. Some ARGs were detected in all environmental samples in relation to the activities of egrets (Jin River water, bird feces, and the park soil) such as *aac6ib* and *aadA*. Some ARGs were only detected in river water and bird feces such as certain tetracycline resistance genes (*tetL* and *tetO*). The others were discovered only in Jin River water such as *aac3ia*, *aac6iia*, and *bla*CMY2. These ARGs were hardly transported among different environmental locations. The abundance of ARGs in the environment declined along the transportation route (i.e., from polluted waterway to egrets and then to the park soil affected by egrets, refer to **Figure 5A**). Most of the ARGs that are detected in the water samples from Jin River are absent (undetectable) in the campus soil. The MGE sequences (four transposase genes and one integrase gene) were also quantified. The abundance of these genes also declined along the AR transportation route. These genes were not detected in the campus soil (**Figure 5A**).

### Detection of *mcr-1* Gene in Environmental *E. coli* Isolates

According to the results of PCR survey of *mcr-1* gene, amplicons with the expected size were obtained from three out of the six colistin-resistant strains. The PCR products were submitted to sequencing and proved to be the *mcr-1* gene. Obtained DNA sequences were deposited into GenBank under accession numbers KY218737 to KY218739. Two of the three *mcr-1*-positive *E. coli* strains were isolated from Jin River water samples, and the other one was isolated from egret feces.

## Discussion

In this research, the common fecal indicator bacterium, *E. coli*, was isolated from environmental samples to assess the bacterial AR of the environment. *E. coli* as an indicator organism is currently used to monitor microbiological water quality and is well characterized in terms of acquired AR (Berendonk et al., 2015). This method may leave out some AR mechanism tied to special bacteria species. But it is still reasonable to a large extent if the ubiquitous HGT is taken into consideration (Rosen et al., 2015). Slight differences in the components of culture broth, the purities and potencies of antibiotics of different suppliers may affect the obtained MIC data. The introduction of the standard strain and the normalization of the MIC data against the MICs of the standard strain to some extend shielded these influences. Resistant breakpoints were set at 16 times of the MICs of the susceptible standard strain (ATCC 25922). The environmental isolates and the standard strain are of the same bacterial species, while the environmental isolates can survive at significantly higher antibiotic concentration. It is reasonable to believe that there must be considerable AR mechanisms in environmental isolates over the standard strain that endows them the capability to survive at the higher antibiotic concentration.

Jin River runs through wide-open irrigated agricultural area before flowing into the urban area of Chengdu. In China, there is no strict prohibition on antibiotic applications in animal husbandry and aquaculture. A dozen kinds of antibiotics are still on the permission list of feed additives. China shares the largest global antimicrobial consumption in food animal production (23% in 2010; Van Boeckel et al., 2015). The open discharge of wastewater from animal husbandry and aquaculture brings serious AR pollution into the water body. As combined sewer system is still in use at parts of the city, the river water carries both the agricultural and urban influences. It is predictable to observe high level of AR in the river. Although the park and the campus are geographically adjacent and are just separated by a road, the Wangjiang Road (**Figure 1**), the AR is significantly higher in the park soil (higher resistance percentages with *p* < 0.01). This probably relates to the influence of the egret on the park soil. According to our knowledge, there is no antibiotic selective pressure artificially introduced to the park soil via either manure fertilization or reclaimed water irrigation. As we did not analyze the antibiotic pollutants in the park soil, the possibility of selective pressure of antibiotics on the soil bacterial community could not be fully excluded. The resistance percentage of *E. coli* isolates against each antibiotic was the highest in river water, and the drug-resistance situation in the egret feces and the park soil was closely related to that in the river water (**Figure 2A**). More quantitatively, the AR rate among the *E. coli* isolates from bird feces and park soil exponentially correlated with the AR rate of the Jin River water isolates (**Figure 2B**). All these facts above suggested that egrets to some extent mediated the environmental transportation of AR.

For the environmental locations in relation to egret’s inhabitation, the percentages of AR in different locations were closely related to each other (**Figure 2B**). Some isolates recovered from different environmental samples did show similar AR patterns (the clusters formed by the points of different environmental samples in **Figure 4**), the number of the cases was, however, limited. More cases were that the bacteria isolated from different environmental samples exhibited distinct AR patterns, and MDR declined along the transportation route. There was no strong correlation between the genotypes and the AR patterns of the resistant *E. coli* isolates (Supplementary Table S7). Especially, the samples of river water and the park soil were associated in time, but the genotypic similarity of *E. coli* isolated from two types of environmental samples was fairly low (Supplementary Figure S1). It was to be expected that *E. coli* strains isolated from different environmental samples gave distinct genotypes. Rep-PCR genotyping had ever been used to track the source of environmental *E. coli*, and fairly good results were obtained (Dombek et al., 2000). The direct transfer of resistant bacteria among different environmental locations seemed rare herein. We cannot fully rule out the possibility of resistant bacteria transferring among environmental locations for the limited sampling scale and the temporal difference among the environmental *E. coli* isolates. Multilocus sequence typing and phylogenetic group designating ever revealed that wild bird and human share ESBL-producing *E. coli* population (Bonnedahl et al., 2009). The results herein, however, suggest that the transfer of resistant bacteria was not the dominant mechanism for the dissemination of AR in environment, at least under the specific transportation mode of AR in this study.

The abundance of ARGs in the environment declined along the transportation route (**Figure 5A**). This could be the reason why the level of MDR declined along the AR transportation route as shown in **Figure 3**. As the source of AR pollution, the Jin River water showed the highest level of ARGs, and as the deliverer and receptor, respectively, egret feces and park soil exhibited comparable abundance of ARGs. The campus soil, however, gave the lowest abundance. Some β-lactamase (i.e., *bla*TEM and *bla*CTX-M-14) and aminoglycoside *O*-phosphotransferase (*aph*) genes should be the indigenous genes of soil bacterial community (Forsberg et al., 2014; Johnson et al., 2016) and were even detected in campus soil. According to a previous study, mobility elements syntenic with ARGs were rare in soil by comparison with sequenced pathogens, suggesting that ARGs may not transfer between soil bacteria as readily as is observed between human pathogens. Therefore, it has been suggested that bacterial community composition primarily determines the soil ARG content and HGT cannot fully decouple the resistome from phylogeny therein (Forsberg et al., 2014). According to our qPCR results, the MGE sequences responsible for HGT such as *tnpA-1* (transposase) and *intI1* (integrase) were enriched in park soil under the effect of egrets in comparison with the campus soil, which implied that MGEs could mediate the transportation of AR therein. ARGs have been observed to co-localize with MGE sequences in the bacterial communities intensively affected by antibiotics (Partridge et al., 2009; Johnson et al., 2016), which drives the horizontal transfer of these ARGs. In our case, the transported ARGs (*aac6ib* and *aadA*) clustered with the MRGs (*intI1* and *tnpA-1*) at the clustering analysis of the abundance data of these genes (**Figure 5B**). We searched the nucleotide database at the National Center for Biotechnology Information (NCBI) to ascertain if there was any precedent for the gene cluster that we observed. An *E. coli* plasmid (AF550679.1) and a *Klebsiella pneumoniae* plasmid (KC958437.1) were found carrying the gene cassettes containing genes *aac6ib*, *intI1*, *tnpA*, and *aadA*. Although various ARGs showed variant transferability, and the internal reasons are yet to be uncovered. The qPCR results presented here provided evidences that HGT contributed to the special transportation mode of environmental AR. The correlation of different ARGs with gene cassettes in relation to genetic mobility need to be clarified via intensive sequencing. As HGT may dominate the environmental transportation of AR, to block HGT can be an efficient method to control the environmental dissemination of AR. How to inhibit the transfer of resistance genes can be a good point of future study.

Colistin is one of the last-resort antibiotics for infection of multidrug-resistant bacteria. Its resistance was ever considered involving chromosomal mutations and cannot be transported among bacteria via HGT. But recently, plasmid-mediated transferable colistin resistance encoded by the gene *mcr-1* was first described in China (Liu et al., 2016) and the discoveries of the same AR gene were reported successively almost all over the rest regions of the world (Skov and Monnet, 2016). China thereafter officially banned colistin as a feed additive for animals on November 1, 2016 (Walsh and Wu, 2016). This is a manifestation of the responsibility of great powers. We had tried to determine the abundance of *mcr-1* in environmental samples via qPCR according to a reported method (Bontron et al., 2016). The abundance of this gene in the environment seems too low to be detected. It was not even detected in the Jin River water samples (data not shown), while we actually encountered several colistin-resistant *E. coli* isolates (*n* = 6). The movable colistin-resistant gene (*mcr-1*) was also detected in three colistin-resistant *E. coli* isolates among which two were isolated from Jin River water and one from egret feces. The genotypes of the three isolates are different from each other according to their rep-PCR banding patterns (*E. coli* isolates W_C08, W_H05, and B_A12 in Supplementary Figure S1). This should be the first case of the simultaneous detection of *mcr-1* positive *E. coli* in wild birds and their habitat, which gave the possibility of environmental transportation of this gene even through birds’ activities. When tracking the source of *mcr-1* gene in previous studies, the stream of people (entry–exit people) and goods (import and export commodities) were closely concerned (Arcilla et al., 2016; Fernandes et al., 2016; Kluytmans-van den Bergh et al., 2016; McGann et al., 2016), while environmental dissemination of this gene was rarely considered and much less the role that migratory birds might play in the process. In central parts of Chile, the ESBL-positive *E. coli* strains were isolated from fecal flora of wild birds (Franklin’s gulls) and the detection rate is even higher than that from local human (Hernandez et al., 2013). In Taif province, Saudi Arabia, MDR was more serious among *Enterobacter* strains isolated from migratory birds than local resident birds (Abo-Amer and Shobrak, 2015). The study in Sweden indicates that a potential of AR transfer between the human population and wild birds exists even in countries with a low level of AR (Bonnedahl et al., 2010). According to the study carried out along the northeastern coast of the United States, AR was more widespread in bacteria isolated from seabirds than those isolated from marine mammals (Rose et al., 2009). Birds, especially the migratory birds, due to their numerous amount and wide range of activities, need to be paid much more attention to on their roles in mediating the transportation of environmental AR. The effective control of AR should be based on universal worldwide strategies. The restraint of antibiotic application in local area cannot create “Peach Blossom Shangri-La” that is free from AR pollution.

## Author Contributions

KY and YZ designed the research. DR isolated *E. coli* from environmental samples. YH collected the MIC data and operated the rep-PCR. JW and DR operated qPCR. JW did the PCR survey of *mcr-1* gene and sent the PCR products for sequencing. YH, JW, and KY treated the data. KY and JW drafted the manuscript, which was edited by YZ.

## Conflict of Interest Statement

The authors declare that the research was conducted in the absence of any commercial or financial relationships that could be construed as a potential conflict of interest.

## References

[B1] Abo-Amer A. E. Shobrak M. Y. (2015). Antibiotic resistance and molecular characterization of *Enterobacter cancerogenus* isolated from wild birds in Taif province, Saudi Arabia. *Thai J. Vet. Med.* 45 101–111.

[B2] Allen H. K. Donato J. Wang H. H. Cloud-Hansen K. A. Davies J. Handelsman J. (2010). Call of the wild: antibiotic resistance genes in natural environments. *Nat. Rev. Microbiol.* 8 251–259. 10.1038/nrmicro2312 20190823

[B3] Allen H. K. Moe L. A. Rodbumrer J. Gaarder A. Handelsman J. (2009). Functional metagenomics reveals diverse beta-lactamases in a remote Alaskan soil. *ISME J.* 3 243–251. 10.1038/ismej.2008.86 18843302

[B4] Andrews J. M. (2001). Determination of minimum inhibitory concentrations. *J. Antimicrob. Chemother.* 48(Suppl. 1), 5–16. 10.1093/jac/48.suppl_1.5 11420333

[B5] Arcilla M. S. van Hattem J. M. Matamoros S. Melles D. C. Penders J. de Jong M. D. (2016). Dissemination of the mcr-1 colistin resistance gene. *Lancet Infect. Dis.* 16 147–149. 10.1016/S1473-3099(15)00541-1 26711361

[B6] Bao X. Qiang Z. Chang J. H. Ben W. Qu J. (2014). Synthesis of carbon-coated magnetic nanocomposite (Fe_3_O_4_@C) and its application for sulfonamide antibiotics removal from water. *J. Environ. Sci.* 26 962–969. 10.1016/S1001-0742(13)60485-4 25079626

[B7] Berendonk T. U. Manaia C. M. Merlin C. Fatta-Kassinos D. Cytryn E. Walsh F. (2015). Tackling antibiotic resistance: the environmental framework. *Nat. Rev. Microbiol.* 13 310–317. 10.1038/nrmicro3439 25817583

[B8] Bhullar K. Waglechner N. Pawlowski A. Koteva K. Banks E. D. Johnston M. D. (2012). Antibiotic resistance is prevalent in an isolated cave microbiome. *PLoS One* 7:e34953. 10.1371/journal.pone.0034953 22509370 PMC3324550

[B9] Boehm A. B. Griffith J. McGee C. Edge T. A. Solo-Gabriele H. M. Whitman R. (2009). Faecal indicator bacteria enumeration in beach sand: a comparison study of extraction methods in medium to coarse sands. *J. Appl. Microbiol.* 107 1740–1750. 10.1111/j.1365-2672.2009.04440.x 19659700 PMC2810257

[B10] Bonnedahl J. Drobni M. Gauthier-Clerc M. Hernandez J. Granholm S. Kayser Y. (2009). Dissemination of *Escherichia coli* with CTX-M type ESBL between humans and yellow-legged gulls in the south of France. *PLoS One* 4:e5958. 10.1371/journal.pone.0005958 19536298 PMC2694269

[B11] Bonnedahl J. Drobni P. Johansson A. Hernandez J. Melhus A. Stedt J. (2010). Characterization, and comparison, of human clinical and black-headed gull (*Larus ridibundus*) extended-spectrum beta-lactamase-producing bacterial isolates from Kalmar, on the southeast coast of Sweden. *J. Antimicrob. Chemother.* 65 1939–1944. 10.1093/jac/dkq222 20615928

[B12] Bontron S. Poirel L. Nordmann P. (2016). Real-time PCR for detection of plasmid-mediated polymyxin resistance (mcr-1) from cultured bacteria and stools. *J. Antimicrob. Chemother.* 71 2318–2320. 10.1093/jac/dkw139 27121402

[B13] Cristóbal-Azkarate J. Dunn J. C. Day J. M. Amabile-Cuevas C. F. (2014). Resistance to antibiotics of clinical relevance in the fecal microbiota of Mexican wildlife. *PLoS One* 9:e107719. 10.1371/journal.pone.0107719 25233089 PMC4169449

[B14] Cui H. Yang K. Pagaling E. Yan T. (2013). Spatial and temporal variation in enterococcal abundance and its relationship to the microbial community in Hawaii beach sand and water. *Appl. Environ. Microbiol.* 79 3601–3609. 10.1128/AEM.00135-13 23563940 PMC3675952

[B15] Dolejska M. Bierosova B. Kohoutova L. Literak I. Cizek A. (2009). Antbiotic-resistant *Salmonella* and *Escherichia coli* isolates with integrons and extended-spectrum beta-lactamases in surface water and sympatric black-headed gulls. *J. Appl. Microbiol.* 106 1941–1950. 10.1111/j.1365-2672.2009.04155.x 19245407

[B16] Dolejska M. Cizek A. Literak I. (2007). High prevalence of antimicrobial-resistant genes and integrons in *Escherichia coli* isolates from Black-headed Gulls in the Czech Republic. *J. Appl. Microbiol.* 103 11–19. 10.1111/j.1365-2672.2006.03241.x 17584448

[B17] Dombek P. E. Johnson L. K. Zimmerley S. T. Sadowsky M. J. (2000). Use of repetitive DNA sequences and the PCR to differentiate *Escherichia coli* isolates from human and animal sources. *Appl. Environ. Microbiol.* 66 2572–2577. 10.1128/AEM.66.6.2572-2577.2000 10831440 PMC110583

[B18] Fernandes M. R. Moura Q. Sartori L. Silva K. C. Cunha M. P. Esposito F. (2016). Silent dissemination of colistin-resistant *Escherichia coli* in South America could contribute to the global spread of the mcr-1 gene. *Euro Surveill.* 21 1–6. 10.2807/1560-7917.ES.2016.21.17.30214 27168587

[B19] Forsberg K. J. Patel S. Gibson M. K. Lauber C. L. Knight R. Fierer N. (2014). Bacterial phylogeny structures soil resistomes across habitats. *Nature* 509 612–616. 10.1038/nature13377 24847883 PMC4079543

[B20] Forsberg K. J. Reyes A. Wang B. Selleck E. M. Sommer M. O. Dantas G. (2012). The shared antibiotic resistome of soil bacteria and human pathogens. *Science* 337 1107–1111. 10.1126/science.1220761 22936781 PMC4070369

[B21] Foti M. Rinaldo D. Guercio A. Giacopello C. Aleo A. De Leo F. (2011). Pathogenic microorganisms carried by migratory birds passing through the territory of the island of Ustica, Sicily (Italy). *Avian Pathol.* 40 405–409. 10.1080/03079457.2011.588940 21812720

[B22] Fukahori S. Fujiwara T. Funamizu N. Matsukawa K. Ito R. (2013). Adsorptive removal of sulfonamide antibiotics in livestock urine using the high-silica zeolite HSZ-385. *Water Sci. Technol.* 67 319–325. 10.2166/wst.2012.513 23168630

[B23] Guenther S. Aschenbrenner K. Stamm I. Bethe A. Semmler T. Stubbe A. (2012). Comparable high rates of extended-spectrum-beta-lactamase-producing *Escherichia coli* in birds of prey from Germany and Mongolia. *PLoS One* 7:e53039. 10.1371/journal.pone.0053039 23300857 PMC3534101

[B24] Hernandez J. Johansson A. Stedt J. Bengtsson S. Porczak A. Granholm S. (2013). Characterization and comparison of extended-spectrum beta-lactamase (ESBL) resistance genotypes and population structure of *Escherichia coli* isolated from Franklin’s gulls (*Leucophaeus pipixcan*) and humans in Chile. *PLoS One* 8:e76150. 10.1371/journal.pone.0076150 24098774 PMC3786981

[B25] Hirsch R. Ternes T. Haberer K. Kratz K. L. (1999). Occurrence of antibiotics in the aquatic environment. *Sci. Total Environ.* 225 109–118. 10.1016/S0048-9697(98)00337-4 10028708

[B26] Homem V. Santos L. (2011). Degradation and removal methods of antibiotics from aqueous matrices–a review. *J. Environ. Manage.* 92 2304–2347. 10.1016/j.jenvman.2011.05.023 21680081

[B27] Ji L. Chen W. Zheng S. Xu Z. Zhu D. (2009). Adsorption of sulfonamide antibiotics to multiwalled carbon nanotubes. *Langmuir* 25 11608–11613. 10.1021/la9015838 19725569

[B28] Johnson T. A. Stedtfeld R. D. Wang Q. Cole J. R. Hashsham S. A. Looft T. (2016). Clusters of antibiotic resistance genes enriched together stay together in swine agriculture. *mBio* 7 e02214–e02215. 10.1128/mBio.02214-15 27073098 PMC4959523

[B29] Kluytmans-van den Bergh M. F. Huizinga P. Bonten M. J. Bos M. De Bruyne K. Friedrich A. W. (2016). Presence of mcr-1-positive *Enterobacteriaceae* in retail chicken meat but not in humans in the Netherlands since 2009. *Euro Surveill.* 21:30149. 10.2807/1560-7917.ES.2016.21.9.30149 26967540

[B30] Krumperman P. H. (1983). Multiple antibiotic resistance indexing of *Escherichia coli* to identify high-risk sources of fecal contamination of foods. *Appl. Environ. Microbiol.* 46 165–170. 6351743 10.1128/aem.46.1.165-170.1983 PMC239283

[B31] Literak I. Vanko R. Dolejska M. Cizek A. Karpiskova R. (2007). Antibiotic resistant *Escherichia coli* and *Salmonella* in Russian rooks (*Corvus frugilegus*) wintering in the Czech Republic. *Lett. Appl. Microbiol.* 45 616–621. 10.1111/j.1472-765X.2007.02236.x 17916127

[B32] Liu Y. Y. Wang Y. Walsh T. R. Yi L. X. Zhang R. Spencer J. (2016). Emergence of plasmid-mediated colistin resistance mechanism MCR-1 in animals and human beings in China: a microbiological and molecular biological study. *Lancet Infect. Dis.* 16 161–168. 10.1016/S1473-3099(15)00424-7 26603172

[B33] Luo Y. Mao D. Rysz M. Zhou Q. Zhang H. Xu L. (2010). Trends in antibiotic resistance genes occurrence in the Haihe River, China. *Environ. Sci. Technol.* 44 7220–7225. 10.1021/es100233w 20509603

[B34] McGann P. Snesrud E. Maybank R. Corey B. Ong A. C. Clifford R. (2016). *Escherichia coli* Harboring mcr-1 and blaCTX-M on a Novel IncF Plasmid: First report of mcr-1 in the USA. *Antimicrob. Agents Chemother.* 60 4420–4421. 10.1128/AAC.01103-16 27230792 PMC4914657

[B35] Muller F. Taylor P. D. Sjoberg S. Muheim R. Tsvey A. Mackenzie S. A. (2016). Towards a conceptual framework for explaining variation in nocturnal departure time of songbird migrants. *Mov. Ecol.* 4:24. 10.1186/s40462-016-0089-2 27833750 PMC5066284

[B36] Partridge S. R. Tsafnat G. Coiera E. Iredell J. R. (2009). Gene cassettes and cassette arrays in mobile resistance integrons. *FEMS Microbiol. Rev.* 33 757–784. 10.1111/j.1574-6976.2009.00175.x 19416365

[B37] Peterson J. W. Petrasky L. J. Seymour M. D. Burkhart R. S. Schuiling A. B. (2012). Adsorption and breakdown of penicillin antibiotic in the presence of titanium oxide nanoparticles in water. *Chemosphere* 87 911–917. 10.1016/j.chemosphere.2012.01.044 22342282

[B38] Poeta P. Radhouani H. Igrejas G. Goncalves A. Carvalho C. Rodrigues J. (2008). Seagulls of the berlengas natural reserve of portugal as carriers of fecal *Escherichia coli* harboring CTX-M and TEM extended-spectrum beta-lactamases. *Appl. Environ. Microbiol.* 74 7439–7441. 10.1128/AEM.00949-08 18835997 PMC2592940

[B39] Radhouani H. Poeta P. Goncalves A. Pacheco R. Sargo R. Igrejas G. (2012). Wild birds as biological indicators of environmental pollution: antimicrobial resistance patterns of *Escherichia coli* and enterococci isolated from common buzzards (Buteo buteo). *J. Med. Microbiol.* 61 837–843. 10.1099/jmm.0.038364-0 22403140

[B40] Radimersky T. Frolkova P. Janoszowska D. Dolejska M. Svec P. Roubalova E. (2010). Antibiotic resistance in faecal bacteria (*Escherichia coli. Enterococcus* spp.) in feral pigeons. *J. Appl. Microbiol.* 109 1687–1695. 10.1111/j.1365-2672.2010.04797.x 20602656

[B41] Rose J. M. Gast R. J. Bogomolni A. Ellis J. C. Lentell B. J. Touhey K. (2009). Occurrence and patterns of antibiotic resistance in vertebrates off the Northeastern United States coast. *FEMS Microbiol. Ecol.* 67 421–431. 10.1111/j.1574-6941.2009.00648.x 19187217 PMC5444207

[B42] Rosen M. J. Davison M. Bhaya D. Fisher D. S. (2015). Microbial diversity. Fine-scale diversity and extensive recombination in a quasisexual bacterial population occupying a broad niche. *Science* 348 1019–1023. 10.1126/science.aaa4456 26023139

[B43] Seifrtova M. Pena A. Lino C. M. Solich P. (2008). Determination of fluoroquinolone antibiotics in hospital and municipal wastewaters in Coimbra by liquid chromatography with a monolithic column and fluorescence detection. *Anal. Bioanal. Chem.* 391 799–805. 10.1007/s00216-008-2020-1 18425644

[B44] Shi L. Ma F. Han Y. Zhang X. Yu H. (2014). Removal of sulfonamide antibiotics by oriented immobilized laccase on Fe_3_O_4_ nanoparticles with natural mediators. *J. Hazard. Mater.* 279 203–211. 10.1016/j.jhazmat.2014.06.070 25064257

[B45] Skov R. L. Monnet D. L. (2016). Plasmid-mediated colistin resistance (mcr-1 gene): three months later, the story unfolds. *Euro Surveill.* 21:30155. 10.2807/1560-7917.ES.2016.21.9.30155 26967914

[B46] Skurnik D. Ruimy R. Andremont A. Amorin C. Rouquet P. Picard B. (2006). Effect of human vicinity on antimicrobial resistance and integrons in animal faecal *Escherichia coli*. *J. Antimicrob. Chemother.* 57 1215–1219. 10.1093/jac/dkl122 16581916

[B47] Su H. C. Ying G. G. Tao R. Zhang R. Q. Fogarty L. R. Kolpin D. W. (2011). Occurrence of antibiotic resistance and characterization of resistance genes and integrons in *Enterobacteriaceae* isolated from integrated fish farms in South China. *J. Environ. Monit.* 13 3229–3236. 10.1039/c1em10634a 21975604

[B48] Su H. C. Ying G. G. Tao R. Zhang R. Q. Zhao J. L. Liu Y. S. (2012). Class 1 and 2 integrons, sul resistance genes and antibiotic resistance in *Escherichia coli* isolated from Dongjiang River, South China. *Environ. Pollut.* 169 42–49. 10.1016/j.envpol.2012.05.007 22683479

[B49] Tao R. Ying G. G. Su H. C. Zhou H. W. Sidhu J. P. (2010). Detection of antibiotic resistance and tetracycline resistance genes in *Enterobacteriaceae* isolated from the Pearl rivers in South China. *Environ. Pollut.* 158 2101–2109. 10.1016/j.envpol.2010.03.004 20356660

[B50] Thaller M. C. Migliore L. Marquez C. Tapia W. Cedeno V. Rossolini G. M. (2010). Tracking acquired antibiotic resistance in commensal bacteria of Galapagos land iguanas: no man, no resistance. *PLoS One* 5:e8989. 10.1371/journal.pone.0008989 20126545 PMC2813872

[B51] USEPA (2002). *Method 1103.1: Escherichia coli (E. coli) in Water by Membrane Filtration Using Membrane-Thermotolerant Escherichia coli Agar (mTEC)*. Washington, DC: United States Environmental Protection Agency.

[B52] Van Boeckel T. P. Brower C. Gilbert M. Grenfell B. T. Levin S. A. Robinson T. P. (2015). Global trends in antimicrobial use in food animals. *Proc. Natl. Acad. Sci. U.S.A.* 112 5649–5654. 10.1073/pnas.1503141112 25792457 PMC4426470

[B53] Walsh T. R. Wu Y. (2016). China bans colistin as a feed additive for animals. *Lancet Infect. Dis.* 16 1102–1103. 10.1016/S1473-3099(16)30329-2 27676338

[B54] Wei R. Ge F. Huang S. Chen M. Wang R. (2011). Occurrence of veterinary antibiotics in animal wastewater and surface water around farms in Jiangsu Province, China. *Chemosphere* 82 1408–1414. 10.1016/j.chemosphere.2010.11.067 21159362

[B55] Xiong W. Sun Y. Zhang T. Ding X. Li Y. Wang M. (2015). Antibiotics, antibiotic resistance genes, and bacterial community composition in fresh water aquaculture environment in China. *Microb. Ecol.* 70 425–432. 10.1007/s00248-015-0583-x 25753824

[B56] Xu W. Zhang G. Li X. Zou S. Li P. Hu Z. (2007). Occurrence and elimination of antibiotics at four sewage treatment plants in the Pearl River Delta (PRD), South China. *Water Res.* 41 4526–4534. 10.1016/j.watres.2007.06.023 17631935

[B57] Yang J. F. Ying G. G. Zhao J. L. Tao R. Su H. C. Chen F. (2010). Simultaneous determination of four classes of antibiotics in sediments of the Pearl Rivers using RRLC-MS/MS. *Sci. Total Environ.* 408 3424–3432. 10.1016/j.scitotenv.2010.03.049 20451241

[B58] Yogui G. T. Sericano J. L. (2009). Levels and pattern of polybrominated diphenyl ethers in eggs of Antarctic seabirds: endemic versus migratory species. *Environ. Pollut.* 157 975–980. 10.1016/j.envpol.2008.10.016 19027208

[B59] Zhang Q. Q. Ying G. G. Pan C. G. Liu Y. S. Zhao J. L. (2015). Comprehensive evaluation of antibiotics emission and fate in the river basins of china: source analysis, multimedia modeling, and linkage to bacterial resistance. *Environ. Sci. Technol.* 49 6772–6782. 10.1021/acs.est.5b00729 25961663

[B60] Zhu Y. G. Johnson T. A. Su J. Q. Qiao M. Guo G. X. Stedtfeld R. D. (2013). Diverse and abundant antibiotic resistance genes in Chinese swine farms. *Proc. Natl. Acad. Sci. U.S.A.* 110 3435–3440. 10.1073/pnas.1222743110 23401528 PMC3587239

[B61] Zou S. Xu W. Zhang R. Tang J. Chen Y. Zhang G. (2011). Occurrence and distribution of antibiotics in coastal water of the Bohai Bay, China: impacts of river discharge and aquaculture activities. *Environ. Pollut.* 159 2913–2920. 10.1016/j.envpol.2011.04.037 21576000

[B62] Zuo L. Ai J. Fu H. Chen W. Zheng S. Xu Z. (2016). Enhanced removal of sulfonamide antibiotics by KOH-activated anthracite coal: batch and fixed-bed studies. *Environ. Pollut.* 211 425–434. 10.1016/j.envpol.2015.12.064 26802515

