# Supplementary material for: Evidence for Environmental Dissemination of Antibiotic Resistance Mediated by Wild Birds
Source: Front Microbiol. 2018 Apr 20;9:745. doi: 10.3389/fmicb.2018.00745 (PMC5921526; doi:10.3389/fmicb.2018.00745)
Supplement: Supplementary file 1 [file Table_1.DOCX]

***Supplementary Material***

**Evidences for environmental dissemination of antibiotic resistance mediated by wild birds**

**Jiao Wu ^#^, Ye Huang^#^, Dawei Rao, Yongkui Zhang, Kun Yang^*^**

Department of Pharmaceutical & Biological Engineering, School of Chemical Engineering, Sichuan University, Chengdu, Sichuan province, China

*** Correspondence:**

Kun Yang

cookyoung@scu.edu.cn

**Table S1.** *E. coli* isolates collections (numbers) on different dates and from different samples.

| **Sampling date** | **Sampling sites (for Jin River water samples) or No. of samples (for other samples)** | | | | |
| --- | --- | --- | --- | --- | --- |
|  | **1** | **2** | **3** | **4** | **5** |
| ***E. coli* isolated form Jin River water samples (n=95)** | | | | | |
| Jan 21 2015 | 5 | 5 | 5 | 5 | 4 |
| Mar 13 2015 | 4 | 4 | 4 | 0 | 0 |
| Mar 26 2015 | 6 | 6 | 6 | 6 | 6 |
| Apr 1 2015 | 6 | 5 | 6 | 6 | 6 |
|  |  |  |  |  |  |
| ***E. coli* isolated from egret feces samples (n=94)** | | | | | |
| Apr 27 2015 | 21 | 17 | 24 | 19 | 13 |
|  |  |  |  |  |  |
| ***E. coli* isolated from Wangjiang Park soil samples (n=81)** | | | | | |
| Jan 21 2015 | 5 | 5 | 5 | 5 | 5 |
| Mar 13 2015 | 8 | 8 | 3 | 0 | 0 |
| Mar 26 2015 | 6 | 6 | 6 | 6 | 0 |
| Apr 1 2015 | 8 | 0 | 0 | 4 | 1 |
|  |  |  |  |  |  |
| ***E. coli* isolated from campus soil samples (n=91)** | | | | | |
| Sep 7 2015 | 5 | 9 | 2 | 2 | 73 |

**Table S2.** Information of primer pairs used in qPCR

| **Genes** | **Related antibiotics** | **Encoding** | **Primer For** | **Primer Rev** | **Product size (bp)** | **Annealing temperature (^o^C)** |
| --- | --- | --- | --- | --- | --- | --- |
| 16S rRNA^a^ | None | 16S rRNA | GGGTTGCGCTCGTTGC | ATGGYTGTCGTCAGCTCGTG | 60 | 59 |
| *tetW* | Tetracycline | Ribosomal protection protein | CCGCTCTTTGGCTGTTTTAG | GCCTGGTCGATCTTGTTGAT | 141 | 53 |
| *tetO* | Tetracycline | Ribosomal protection protein | CTTTCGGCTGCTTTCCCCTA | TGGAGCATCATGATACGCCC | 133 | 56 |
| *tetL* | Tetracycline | Major facilitator superfamily transporter, tetracycline efflux pump | CTGCATTTCCAGCACTCGTA | ATGGGCTATCATTCCACCAA | 140 | 56 |
| *aph33ib*  *aph6id* | Streptomycin | Aminoglycoside O-phosphotransferase | TATGGTTGTTTGCCATGGTG | CCAGTTCTCTTCGGCGTTAG | 154 | 59 |
| *aac6ib* | Amikacin | Aminoglycoside N-acetyltransferase | TCCAGGAGTACGCGGAATAG | CGTTTGGATCTTGGTGACCT | 130 | 59 |
| *aac3ia* | Gentamicin | Aminoglycoside N-acetyltransferase | CATCATTCGCACATGTAGGC | CAAGCGCGATGAATGTCTTA | 164 | 59 |
| *aac6iia* | Gentamicin | Aminoglycoside N-acetyltransferase | CACCGTGACGAAGATTCAGA | TTCTCTCGAAGGCTTGTCGT | 152 | 56 |
| *blaTEM* | cephalosporin; penicillin | Beta-lactamase | AAGCCATACCAAACGACGAG | AACTTTATCCGCCTCCATCC | 137 | 59 |
| *aadA^a^* | Kanamycin | Aminoglycoside-3'-adenylyltransferase | CGAGATTCTCCGCGCTGTA | GCTGCCATTCTCCAAATTGC | 101 | 62 |
| *blaCMY-2^a^* | Beta-lactam | Beta-lactamase | GCGAGCAGCCTGAAGCA | CGGATGGGCTTGTCCTCTT | 146 | 59 |
| *BlaCTX-M-14*^a^ | Beta-lactam | Beta-lactamase | GGAGGCGTGACGGCTTTT | TTCAGTGCGATCCAGACGAA | 63 | 59 |
| *tnpA-1*^a^ | None | Transposase | CATCATCGGACGGACAGAATT | GTCGGAGATGTGGGTGTAGAAAGT | 113 | 56.5 |
| *IS613*^a^ | None | Transposase | AGGTTCGGACTCAATGCAACA | TTCAGCACATACCGCCTTGAT | 101 | 56.5 |
| *tnpA-2*^a^ | None | Transposase | GGGCGGGTCGATTGAAA | GTGGGCGGGATCTGCTT | 108 | 56.5 |
| *Tp614*^a^ | None | Transposase | GGAAATCAACGGCATCCAGTT | CATCCATGCGCTTTTGTCTCT | 101 | 56.5 |
| *intI1*^b^ | None | Integrase gene of class 1 integrons | CCTCCCGCACGATGATC | TCCACGCATCGTCAGGC | 280 | 56.5 |

a: These primer-pairs were extracted from references (Zhu et al., 2013; Wang et al., 2014; Su et al., 2015).

b: The primer-pair targeting integrase gene was got from references (Goldstein et al., 2001; Chen and Zhang, 2013).

**Table S3.** MICs distribution (%) of Jin River *E. coli* isolates (n=95).

| **Conc. Range (µg/mL)** | **≤0.25** | **0.25-0.5** | **0.5-1** | **1-2** | **2-4** | **4-8** | **8-16** | **16-32** | **32-64** | **64-128** | **>128** | **MICs for ATCC 25922 (µg/mL)** |
| --- | --- | --- | --- | --- | --- | --- | --- | --- | --- | --- | --- | --- |
| Kanamycin A | 0.00 | 1.05 | 1.05 | 0.00 | 0.00 | 2.11 | 18.95 | **43.16** | 9.47 | 2.11 | **22.11** | 8 |
| Amikacin | 0.00 | 0.00 | 0.00 | 1.05 | 10.53 | 55.79 | 29.47 | 3.16 | 0.00 | 0.00 | 0.00 | 4 |
| Gentamicin | 0.00 | 0.00 | 0.00 | 25.26 | **34.74** | 11.58 | 0.00 | 2.11 | 1.05 | 7.37 | **17.89** | 4 |
| Streptomycin | 0.00 | 0.00 | 0.00 | 1.05 | 0.00 | 1.05 | 25.26 | **27.37** | 5.26 | 5.26 | **34.74** | 16 |
| Tetracycline | 0.00 | 1.05 | **15.79** | 10.53 | 0.00 | 0.00 | 1.05 | 2.11 | 17.89 | **26.32** | 25.26 | 2 |
| Cefalexin | 0.00 | 0.00 | 0.00 | 0.00 | 0.00 | 0.00 | 2.11 | **12.63** | 5.26 | 10.53 | **69.47** | 16 |
| Ampicillin | 0.00 | 7.37 | **13.68** | 10.53 | 0.00 | 0.00 | 1.05 | 0.00 | 4.21 | 11.58 | **51.58** | 4 |
| Colistin | 0.00 | 3.16 | 1.05 | 8.42 | 18.95 | 34.74 | 25.26 | 8.42 | 0.00 | 0.00 | 0.00 | 2 |
| Nalidixic acid | 0.00 | 0.00 | 2.11 | 10.53 | **15.79** | 7.37 | 6.32 | 2.11 | 3.16 | 7.37 | **45.26** | 2 |
|  |  |  |  |  |  |  |  |  |  |  |  |  |
| **Conc. Range (µg/mL)** | **≤0.0125** | **0.0125-0.025** | **0.025-0.05** | **0.05-**  **0.1** | **0.1-0.2** | **0.2-0.4** | **0.4-0.8** | **0.8-1.6** | **1.6-3.2** | **3.2-6.4** | **>6.4** | **MICs for ATCC 25922 (µg/mL)** |
| Ceftriaxone | 0.00 | 3.16 | 12.63 | **20.00** | 7.37 | 4.21 | 1.05 | 3.16 | 1.05 | 1.05 | **46.32** | 0.05 |
| Ciprofloxacin | 0.00 | 1.05 | 5.26 | **9.47** | 1.05 | 2.11 | 1.05 | 4.21 | 2.11 | 5.26 | **68.42** | 0.0125 |

**Table S4.** MICs distribution (%) of egret feces *E. coli* isolates (n=94).

| **Conc. Range (µg/mL)** | **≤0.25** | **0.25-0.5** | **0.5-1** | **1-2** | **2-4** | **4-8** | **8-16** | **16-32** | **32-64** | **64-128** | **>128** |
| --- | --- | --- | --- | --- | --- | --- | --- | --- | --- | --- | --- |
| Kanamycin A | 0.00 | 0.00 | 0.00 | 0.00 | 0.00 | 0.00 | 6.38 | **77.66** | 8.51 | 2.13 | **5.32** |
| Amikacin | 0.00 | 0.00 | 0.00 | 0.00 | 1.06 | 0.00 | 7.45 | 90.43 | 1.06 | 0.00 | 0.00 |
| Gentamicin | 0.00 | 0.00 | 0.00 | 1.06 | 21.28 | **72.34** | 4.26 | 0.00 | 0.00 | **1.06** | 0.00 |
| Streptomycin | 0.00 | 0.00 | 0.00 | 0.00 | 0.00 | 1.06 | 1.06 | **64.89** | 2.13 | 11.70 | **19.15** |
| Tetracycline | 0.00 | 0.00 | 25.53 | **36.17** | 0.00 | 0.00 | 1.06 | 0.00 | 9.57 | **18.09** | 9.57 |
| Cefalexin | 0.00 | 0.00 | 0.00 | 0.00 | 0.00 | 0.00 | 0.00 | **38.30** | **39.36** | 9.57 | **12.77** |
| Ampicillin | 0.00 | 0.00 | 10.64 | **29.79** | 12.77 | 1.06 | 2.13 | 1.06 | 1.06 | 3.19 | **38.30** |
| Colistin | 0.00 | 2.13 | 21.28 | 43.62 | 29.79 | 2.13 | 1.06 | 0.00 | 0.00 | 0.00 | 0.00 |
| Nalidixic acid | 0.00 | 1.06 | 18.09 | **31.91** | 11.70 | **20.21** | 9.57 | 2.13 | 0.00 | 2.13 | **3.19** |
|  |  |  |  |  |  |  |  |  |  |  |  |
| **Conc. Range (µg/mL)** | **≤0.0125** | **0.0125-0.025** | **0.025-0.05** | **0.05-0.1** | **0.1-0.2** | **0.2-0.4** | **0.4-0.8** | **0.8-1.6** | **1.6-3.2** | **3.2-6.4** | **>6.4** |
| Ceftriaxone | 2.13 | **25.53** | 10.64 | **42.55** | 9.57 | 4.26 | 0.00 | 0.00 | 0.00 | 0.00 | **5.32** |
| Ciprofloxacin | **51.06** | 9.57 | 2.13 | 3.19 | 12.77 | **14.89** | 0.00 | 3.19 | 0.00 | 0.00 | **3.19** |

**Table S5.** MICs distribution (%) of park soil *E. coli* isolates (n=81).

| Conc. Range (µg/mL) | ≤0.25 | 0.25-0.5 | 0.5-1 | 1-2 | 2-4 | 4-8 | 8-16 | 16-32 | 32-64 | 64-128 | >128 |
| --- | --- | --- | --- | --- | --- | --- | --- | --- | --- | --- | --- |
| Kanamycin A | 0.00 | 0.00 | 0.00 | 0.00 | 0.00 | 12.35 | **64.20** | 16.05 | 0.00 | 0.00 | **7.41** |
| Amikacin | 0.00 | 1.23 | 0.00 | 0.00 | 1.23 | 8.64 | 61.73 | 25.93 | 1.23 | 0.00 | 0.00 |
| Gentamicin | 0.00 | 0.00 | 1.23 | 22.22 | 62.96 | 13.58 | 0.00 | 0.00 | 0.00 | 0.00 | 0.00 |
| Streptomycin | 0.00 | 0.00 | 0.00 | 0.00 | 0.00 | 1.23 | 20.99 | **48.15** | 2.47 | 6.17 | **20.99** |
| Tetracycline | 0.00 | 0.00 | 0.00 | 0.00 | 0.00 | 0.00 | 0.00 | **29.63** | 14.81 | 0.00 | **55.56** |
| Cefalexin | 0.00 | 0.00 | 0.00 | 0.00 | 0.00 | **1.23** | 0.00 | 0.00 | 17.28 | **50.62** | 30.86 |
| Ampicillin | 0.00 | 0.00 | 0.00 | 0.00 | 0.00 | 23.46 | **44.44** | 7.41 | 0.00 | 3.70 | **20.99** |
| Colistin | 58.02 | 24.69 | 3.70 | 8.64 | 3.70 | 1.23 | 0.00 | 0.00 | 0.00 | 0.00 | 0.00 |
| Nalidixic acid | 0.00 | 0.00 | 1.23 | 7.41 | **44.44** | 24.69 | 3.70 | 1.23 | 0.00 | 3.70 | **13.58** |
|  |  |  |  |  |  |  |  |  |  |  |  |
| Conc. Range (µg/mL) | ≤0.0125 | 0.0125-0.025 | 0.025-0.05 | 0.05-0.1 | 0.1-0.2 | 0.2-0.4 | 0.4-0.8 | 0.8-1.6 | 1.6-3.2 | 3.2-6.4 | >6.4 |
| Ceftriaxone | 0.00 | **62.96** | 13.58 | 11.11 | 1.23 | 0.00 | 1.23 | **6.17** | 0.00 | 0.00 | **3.70** |
| Ciprofloxacin | 0.00 | 0.00 | 2.47 | 7.41 | **46.91** | 6.17 | 2.47 | 7.41 | 0.00 | **14.81** | 12.35 |

**Table S6.** MICs distribution (%) of campus soil *E. coli* isolates (n=91).

| Conc. Range (µg/mL) | <=0.25 | 0.25-0.5 | 0.5-1 | 1-2 | 2-4 | 4-8 | 8-16 | 16-32 | 32-64 | 64-128 | >128 |
| --- | --- | --- | --- | --- | --- | --- | --- | --- | --- | --- | --- |
| Kanamycin A | 0.00 | 0.00 | 0.00 | 1.10 | 0.00 | 1.10 | **61.54** | 35.16 | 0.00 | 0.00 | **1.10** |
| Amikacin | 0.00 | 0.00 | 1.10 | 0.00 | 0.00 | 12.09 | 82.42 | 4.40 | 0.00 | 0.00 | 0.00 |
| Gentamicin | 0.00 | 0.00 | 0.00 | 2.20 | 70.33 | 27.47 | 0.00 | 0.00 | 0.00 | 0.00 | 0.00 |
| Streptomycin | 0.00 | 0.00 | 0.00 | 0.00 | 0.00 | 0.00 | 97.80 | 0.00 | 0.00 | 2.20 | 0.00 |
| Tetracycline | 0.00 | 0.00 | **95.60** | 1.10 | 0.00 | 0.00 | 0.00 | 0.00 | 0.00 | 1.10 | **2.20** |
| Cefalexin | 0.00 | 0.00 | 0.00 | 0.00 | 0.00 | 0.00 | 0.00 | 87.91 | 2.20 | 9.89 | 0.00 |
| Ampicillin | 0.00 | 0.00 | 1.10 | **70.33** | 21.98 | 3.30 | 0.00 | **3.30** | 0.00 | 0.00 | 0.00 |
| Colistin | 0.00 | 0.00 | 84.62 | 13.19 | 2.20 | 0.00 | 0.00 | 0.00 | 0.00 | 0.00 | 0.00 |
| Nalidixic acid | 0.00 | 0.00 | 0.00 | 5.49 | 1.10 | 38.46 | 54.95 | 0.00 | 0.00 | 0.00 | 0.00 |
|  |  |  |  |  |  |  |  |  |  |  |  |
| Conc. Range (µg/mL) | <=0.0125 | 0.0125-0.025 | 0.025-0.05 | 0.05-0.1 | 0.1-0.2 | 0.2-0.4 | 0.4-0.8 | 0.8-1.6 | 1.6-3.2 | 3.2-6.4 | >6.4 |
| Ceftriaxone | 4.40 | **84.62** | 2.20 | 0.00 | 0.00 | 0.00 | 1.10 | **3.30** | **3.30** | 1.10 | 0.00 |
| Ciprofloxacin | 5.49 | **81.32** | 1.10 | 0.00 | 2.20 | **9.89** | 0.00 | 0.00 | 0.00 | 0.00 | 0.00 |

**Table S7.** Antibiotics resistance spectrum of *E. coli* isolated from different environmental samples with similar genotypes.

| Cluster ID (Pearson correlation) | Bacterial ID* | Kan. | Ami. | Gen. | Str. | Tet. | Cefal. | Ceftr. | Amp. | Col. | Cip. | Nal. acid |
| --- | --- | --- | --- | --- | --- | --- | --- | --- | --- | --- | --- | --- |
| A (>91.84%) | B_C01 | - | - | - | - | - | - | - | - | - | - | - |
|  | S_G10 | - | - | - | - | - | - | - | - | - | - | - |
| B (>92.53%) | B_C03 | - | - | - | - | - | - | - | - | - | - | - |
|  | B_D03 | - | - | - | - | - | - | - | - | - | - | - |
|  | B_B03 | - | - | - | - | - | - | - | - | - | - | R |
|  | B_E03 | - | - | - | - | - | - | - | - | - | - | - |
|  | B_C05 | - | - | - | - | - | - | - | - | - | - | - |
|  | S_E06 | - | - | - | - | R | R | - | R | - | R | - |
| C (>90.35%) | W_A12 | - | - | - | R | R | R | - | - | - | R | - |
|  | S_B07 | - | - | - | - | R | - | - | - | - | - | - |
| D (>93.71%) | S_H12 | - | - | - | - | - | - | - | - | - | - | - |
|  | S_B03 | - | - | - | R | R | R | R | R | - | - | - |
|  | B_E05 | - | - | - | - | - | - | - | R | - | - | - |
|  | B_F05 | - | - | - | - | - | - | - | R | - | - | - |
|  | B_H05 | - | - | - | - | - | - | - | - | - | - | - |
| E (>92.82%) | W_F12 | - | - | - | - | R | R | - | R | - | R | - |
|  | S_C07 | - | - | - | R | R | R | - | - | - | R | R |
|  | S_C03 | - | - | - | - | R | - | - | - | - | - | - |
| F  (>90.72%) | W_F09 | R | - | - | R | R | R | R | R | - | R | R |
|  | W_H09 | - | - | - | R | R | R | - | - | - | R | - |
|  | W_G09 | R | - | - | R | R | R | R | R | - | R | R |
|  | W_H08 | R | - | - | R | R | R | R | R | - | R | R |
| G  (>90.42%) | S_A06 | - | - | - | R | R | R | - | R | - | - | - |
|  | S_B06 | - | - | - | - | R | - | - | - | - | - | - |
|  | S_B10 | - | - | - | - | - | - | - | - | - | R | R |
|  | S_G09 | - | - | - | R | - | - | - | - | - | - | - |
|  | S_A01 | - | - | - | - | - | - | - | - | - | - | - |
|  | S_B08 | - | - | - | - | - | - | - | - | - | - | - |
|  | S_A02 | R | R | - | - | R | R | R | R | R | R | R |
| H  (>92.00%) | B_H06 | - | - | - | - | - | - | - | - | - | - | - |
|  | B_C07 | - | - | - | - | - | - | - | - | - | - | - |
|  | B_E07 | - | - | - | - | R | - | - | R | - | R | - |
|  | B_F07 | - | - | - | - | R | - | - | R | - | R | - |
|  | B_B10 | - | - | - | - | - | - | - | - | - | - | - |
|  | B_C10 | - | - | - | - | - | - | - | - | - | - | - |
|  | B_D10 | - | - | - | - | - | - | - | - | - | - | - |
|  | B_B05 | - | - | - | - | - | - | - | - | - | - | - |

* The first letter in the bacterial ID indicates the sample where the bacterium was isolated, i.e. **B** for bird feces, **W** for Jin River water and **S** for park soil. The following letter with number means the location on 96 well plates.

**R** means resistant.

**Caption to Figure S1**. Dendrogram showing the genetic relatedness of *E. coli* isolates from Jin River water (marked in green in the color bar on the right side), bird feces (blue) and park soil (red) based on their rep-PCR DNA fingerprints. The red (90% identity) and blue (80% identity) vertical lines indicate the cutoff value for identifying unique genotypes. Bacterial IDs have been assigned to corresponding clades in the dendrogram.

**Appendix 1. Illustration and discussion for qPCR and related data treatments**

**Illustration of primer pairs acquisition and qPCR operation.** The primer pairs targeting special ARGs were obtained from both de novo design and reference to previous studies. For de novo design, reference sequences of certain ARG were downloaded from Antibiotic Resistance Genes Database (<http://ardb.cbcb.umd.edu>) and National Center for Biotechnology Information (NCBI). Thereafter the sequences of same ARG were imported into Mega 6.0 for alignment and found out the conserved sequence. The primer design for each ARG was based on the conserved sequence via the web interface of primer design program, Primer3Plus (<http://www.primer3plus.com>). The designed primer pairs were double checked via Primer-BLAST (NCBI). After in-silico design, certified primer sequences were sent to Sangon Biotech Co. Ltd (Shanghai) for synthesis. Obtained primer pairs were still detected via preliminary PCR experiments to determine the optimum compositions of PCR mixture and annealing temperatures. The quality (/specificity) of the PCR products was verified via gel electrophoresis. The information of all used primer pairs were collected in Table S2. The qPCRs were performed on a qTOWER 2.2 system (Analytik Jena, Jena, Germany). The 20 μL qPCR mixtures contained 10 μL of Platinum SYBR Green qPCR SuperMix (Invitrogen Corporation, Carlsbad, USA), 0.125 μM each primer, and 0.4 mg/mL bovine serum albumin (BSA). Typical qPCR thermo-cycle consisted of an initial denaturation step at 95 °C for 5 min followed by 45 cycles of 95 °C for 30 s, annealing at certain temperature for 30 s and 72 °C for 30 s, and the fluorescence intensity was detected at the end of each thermo cycle. All reactions were run in triplicate. Only samples with at least two replicates that had specific amplification were regarded as positive. The values used in quantification were the averages of replicates or triplicates.

**Data treatment.** The SYBR Green real-time PCR technique was applied. To quantify the abundance of each ARG in environmental samples, the following equation was used.

$A=\frac{C_{0,t}}{C_{0,s}}=\frac{S_{s}{\eta_{s}}^{C_{T,s}}}{S_{t}{\eta_{t}}^{C_{T,t}}}$ (s1)

Where *A* is the abundance of target ARG; *C_0_* the absolute abundance of target genes in the environmental sample (copy number/mL or g sample); *η* the qPCR apparent efficiency of amplification, which is around the value of 2 and can be determined with the amplification curve using the following equation (s3). *S* is the size of amplicon; *C_T_* the threshold cycle number of qPCR. The subscript "s" means the standard reference gene (in our case the 16S rRNA gene), and the subscript "t" means the target ARG. The mechanism guide the qPCR can be described with the following equation.

$F=\phi S_{t}C_{0,t}{\eta_{t}}^{C_{F,t}}=\phi S_{s}C_{0,s}{\eta_{s}}^{C_{F,s}}$ (s2)

Rewriting it into logarithmic form:

$logF=C_{F,t}log\eta_{t}+log\phi S_{t}C_{0,t}=C_{F,s}log\eta_{s}+log\phi S_{s}C_{0,s}$ (s3)

Where *F* is the fluorescence intensity during the stage of exponential amplification; *Φ* the fluorescence efficiency, a constant that is independent of the abundance of target gene and the size of amplicon; *C_F_* the cycle number when fluorescence intensity reaches *F*. The logarithmic fluorescence intensity (*logF*) is linearly related to the cycle number *C_F_*, and the slope of the line is the logarithm of apparent amplification efficiency *η*. The apparent amplification efficiency of each PCR reaction was thus determined.

**Brief discussion on qPCR data treatment.** To quantify the target genes via quantitative real-time PCR and give its relative abundance in reference to a standard gene has already become a general method. When the apparent amplification efficiencies of the target gene and reference gene are comparable. The relative abundance of the target gene can be simply expressed as the difference between the threshold cycle numbers (*C_T_*) of target and reference genes (*C_T,S_*-*C_T,t_*). This is also the universal method to determine the abundance of environmental ARGs.(Schmittgen and Livak, 2008; Zhu et al., 2013; Wang et al., 2014) But, when the discrepancy between the amplification efficiencies of target and reference genes is not negligible, the minor discrepancy on amplification efficiency *η* will induce obvious discriminate in the abundance determined due to the exponential amplification effect.

It should be closely noted that several factors could influence the amplification efficiency of PCR such as the purity of DNA extract of environmental samples and the impurities it contains, the composition of the PCR reagent and the content of each component, and the performance of the Taq enzyme. The exponential amplification effect will magnify these influences. So, the discrepancy of amplification efficiency between different target genes should not be neglected, especially for the research of great geographical span where there are significant differences in sample properties between environmental locations. The apparent PCR amplification efficiency of each gene should be determined with its amplification curve (Equation (s3)) beforehand. Thereafter the apparent amplification efficiency η is substituted in Equation (s1) to calculate the abundance of each target gene. When using the SYBR Green technique, the product molecule size should also be considered when there is obvious difference in PCR product size of target (*S_t_*) and reference (*S_s_*) genes. The abundance calculated with Equation (s1) has already ruled out the effects brought into by the types of environmental samples, the DNA extraction methods, the quantum efficiency of the fluorescence agent, the size of PCR products, or the efficiency of PCR reaction. This can realize the comparison between environmental samples obtained from different space and at different times. Furthermore, if the same data analysis process was used, the results obtained in different lab, using different method of DNA extraction, using qPCR machine of different version and even applying different primer pairs targeting the same gene, should be still intercomparable.

**References**

Chen, H., and Zhang, M.M. (2013). Occurrence and removal of antibiotic resistance genes in municipal wastewater and rural domestic sewage treatment systems in eastern China. *Environment International* 55**,** 9-14.

Goldstein, C., Lee, M.D., Sanchez, S., Hudson, C., Phillips, B., Register, B., et al. (2001). Incidence of class 1 and 2 integrases in clinical and commensal bacteria from livestock, companion animals, and exotics. *Antimicrobial Agents and Chemotherapy* 45(3)**,** 723-726.

Schmittgen, T.D., and Livak, K.J. (2008). Analyzing real-time PCR data by the comparative C(T) method. *Nat Protoc* 3(6)**,** 1101-1108.

Su, J.Q., Wei, B., Ou-Yang, W.Y., Huang, F.Y., Zhao, Y., Xu, H.J., et al. (2015). Antibiotic resistome and its association with bacterial communities during sewage sludge composting. *Environ Sci Technol* 49(12)**,** 7356-7363. doi: 10.1021/acs.est.5b01012.

Wang, F.H., Qiao, M., Su, J.Q., Chen, Z., Zhou, X., and Zhu, Y.G. (2014). High throughput profiling of antibiotic resistance genes in urban park soils with reclaimed water irrigation. *Environ Sci Technol* 48(16)**,** 9079-9085. doi: 10.1021/es502615e.

Zhu, Y.G., Johnson, T.A., Su, J.Q., Qiao, M., Guo, G.X., Stedtfeld, R.D., et al. (2013). Diverse and abundant antibiotic resistance genes in Chinese swine farms. *Proc Natl Acad Sci U S A* 110(9)**,** 3435-3440. doi: 10.1073/pnas.1222743110
